# Properties of a Transport Instrument for Measuring Psychological Impacts of Delay on Commuters, Mokken Scale Analysis

**DOI:** 10.3389/fpsyg.2021.748899

**Published:** 2021-12-14

**Authors:** Mahdi Rezapour, Cristopher Veenstra, Kelly Cuccolo, F. Richard Ferraro

**Affiliations:** ^1^Independent Researcher, Mashhad, Iran; ^2^Department of Psychology, University of North Dakota, Grand Forks, ND, United States

**Keywords:** Mokken scaling, item response theory, instrument, stress, transportation delay

## Abstract

This study assessed the validity of instrument including various negative psychological and physical behaviors of commuters due to the public transport delay. Instruments have been mostly evaluated by parametric method of item response theory (IRT). However, the IRT has been characterized by some restrictive assumptions about the data, focusing on detailed model fit evaluation. The Mokken scale analysis (MSA), as a scaling procedure is a non-parametric method, which does not require adherence to any distribution. The results of the study show that in most regards, our instrument meets the minimum requirements highlighted by the MSA. However, the instrument did not adhere to the minimum requirements of the “scalability” for two variables including “stomach pain” and “increased heart rate”. So, modifications were proposed to address the violations. Although MSA technique has been used frequently in other fields, this is one of the earliest studies to implement the technique in the context of transport psychology.

## Introduction

After finalizing the data collection and before conducting any statistical analysis, it is important to ensure that the instrument adheres to the fundamental properties needed for the measurement. This can be achieved through parametric method such as of item response theory (IRT) or Mokken scale analysis (MSA). In parametric form IRT, the shape of item response function (IRF) is clarified by some algebraic forms, being based on difficulty and discrimination. The limitation of the parametric approach of the IRT is too far-fetched, and the inference and estimation are too pretentions and intricate for the information that they provide (Mokken, 2011). The MSA has often been used to address the shortcomings of the IRT; the MSA does not need to adhere to specific shapes such as two parameters logistic regression like parametric IRT. In other words, the MSA is viewed as a non-parametric approach to IRT, having less strict assumptions than the parametric method of IRT models.

MSA models are based on three main assumptions that can be characterized as: (1) monotonicity, e.g., with an increase in the person’s location on latent variable, the probability of correct answer does not decrease, (2) unidimensionality, where the response reflects evidence for only a single latent variable, and (3) local independencies, where response to an item is not impacted by responses to any other item. Although the MSA has been applied to a wide domain, its application in the context of transport psychology is less common.

The goal of this study was to determine if the instrument conforms to the required characteristics highlighted by the MSA. The first subsection presents studies regarding the delay of a public transport while the second subsection discusses the samples and instrument.

### Psychological Impacts of a Public Transport Delay

While this study focused on evaluating the instrument, it is also important to review other studies that considered transport delay. The impact of delay was evaluated in perceived importance of a real-time information (Rezapour and Ferraro, 2021). The structural equation modeling (SEM) was employed. The results found that three latent factors need to be considered, including motion sickness, psychological and physiological feelings. Also, the study concluded that the relationship between motion sickness and passenger’s information system (PIS) is not by a direct link but linked through mediation of physiological factors.

In another study, the impact of delay on commuters’ psychological feelings on perceived quality of a rail transport was evaluated (Rezapour and Richard Ferraro, 2021). The ordered mixed model was employed. The results showed while feelings of being tired and nervous are random predictors of the perceived quality of the transport, interaction terms between age and gender should be considered.

The importance of stress and mental health has also been highlighted in past transport studied from different perspectives. For instance, a study investigated the pattern of work-related stress in city bus drivers (Useche et al., 2018). The complex relationship between stress and bus drivers’ incidents were investigated. In another study, the impact of adverse mental health condition of drivers was evaluated (Alonso et al., 2017). The study found that headaches, drowsiness, and various negative emotions are some of factors that impaired driver’s ability to operate the bus.

The discussed studies highlight the complex nature of factors associated with delay and translated effect of delay as stress. Thus, in this study it is important to evaluate the validity of the instrument to make sure the instrument has the characteristics needed to be incorporated in the future study.

### Study Importance

Despite the importance of instrument evaluation, the majority of past studies only employed the traditional Cronbach alpha method, and a single study employed the IRT in the field of transportation (Rezapour et al., 2021). So a comprehensive application of MSA is missing from the past studies in the context of transport. Also, it is necessary to explain why the application of the MSA is worthwhile.

The instrument related to transport delay is complex and so MSA could be employed to assess the assumptions needed to measure the instrument validity. Implementation of the MSA is especially important as it provides measurement for items with respect to attributes related to collected information. Although, the IRT might seem to be suitable, it is more restrictive as weak items might end up into a scale. That is a reason that one should make a decision to include an item even one establishes that the IRT model fit the data well (Sijtsma and van der Ark, 2017).

Despite the importance of evaluating instruments, the authors have only found a single study conducted with the help of IRT (Rezapour et al., 2021) in the context of transportation, while no study has employed the MSA technique in the context of transport psychology. Implementation of the MSA is important due to the possible limitations of the IRT. So, this study contributes to the body of knowledge by evaluating the applicability of the MSA in the context of transport.

## Sample and Instruments

This section is presented in two subsections. The first subsection outlines the design of the instrument while the second subsection gives an overview of various instrument’s explanatory variables.

### The Instrument

For the instrument, the commuters were asked to indicate to what degree they agree that they experience various emotional or physical feelings, while facing the delay of the rail transport. The instruments were distributed to 419 commuters at the station of Serdang, which is one of the main stations of Keretapi Tanah Melayu (KTM) in Malaysia. Among all distributed questionnaires, 396 of them were completed and used for the analysis (a response rate of 94%). No imputation was done, and all incomplete questions were removed from the analysis. The surveys were distributed during off-peak hours from 4 pm to 7 pm to be consistent in our evaluation. Instruments were translated into local language, Malay, by a Malaysian Ph.D. student in the field of education. The instrument had an introduction explaining the objective of the study and various sections of the instruments that the commuters were expected to answer. The respondents were informed that they could leave the instrument blank for any reason.

The instrument had two main parts: psychological effects (4 questions) and physical effects (14 questions). All questions were based on the 5–scale question type. It was noted that due to satisfying behavior of respondent, some of the responses might result in incomplete or biased information retrieval (e.g., choosing the first response alternative), or no information retrieval (Krosnick, 1991). A solution has been proposed by giving an alternative of “I do not know” or “undecided” instead of reporting an opinion. As a result, we incorporated an alternative response of “undecided” in all instruments and questions, except for the first part, which included general questions such as gender. This answer could be considered similar to a middle response (Groothuis and Whitehead, 2002). However, it should be noted that the challenges for including “undecided” have also noted such as conceptual midpoint to one side of the visual midpoint might increase the proportion of respondent choosing a response from the opposite side (Tourangeau et al., 2004) and thus skewing the response.

To evaluate the feelings that the commuters might experience due to delay, the respondent were asked questions in the instrument. For instance, one entry says, “I feel angry when I face delay in KTM” and the respondent responded with their level of agreement on a scale from 1 to 5, 1 meaning they strongly agree and 5 meaning they strongly disagree.

The physical section of the instrument was based on Cohen-Hoberman inventory of physical symptoms (CHIPS) (Cohen and Hoberman, 1983). This is a list of 39 common physical symptoms, and it highlights the relationship between negative life stress and various physical symptomatology. This includes factors such as back pain, diarrhea, and headache.

**FIGURE 1:**
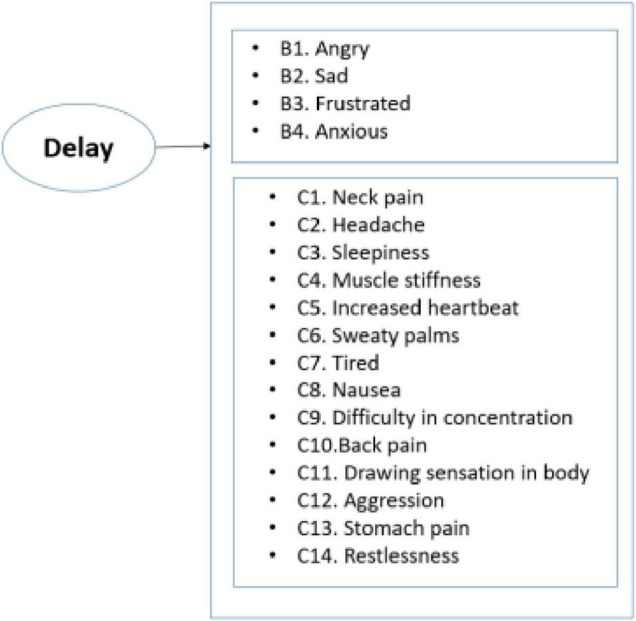
Summary of main predictors in the instrument.

Various sources were used for the design of psychological aspects of instrument. For instance, most of the questions related to the self-report measure of stress were developed and tested in the literature (Greller and Parsons, 1988). The scale included various physiological and psychological descriptors. Psychological factors include factors such as being angry, nervous, or stressed. Some of the physical factors included neck pain and feeling tired. The design of this part of the survey was also similar to the previous study, which was conducted to illustrate the capability of a cognitive-motivational-relational theory for predicting emotions (Lazarus, 1991). For that study, 15 different emotions were identified including negative emotions such as anger, anxiety, sadness, and disgust. The question included in the instrument is depicted in Figure 1.

### Data Descriptions

A total of 396 fully completed responses were collected and considered for the analysis. The respondents were asked about the feelings that they might experience while facing a delay through a 5-point Likert response. The scale had the following alternatives: Very true (as 1), true (2), undecided (3), not true (4), very untrue (5). As shown in Table 1, being frustrated and angry were some of the feelings that respondents most agree they experience while facing a delay in the rail transport. The initial examination of the data reveals that, as expected, an overwhelming majority of the respondents rated the impact of delay very negatively and favor of various emotional or physical feelings. More details about the statistics can be found in Table 2.

**TABLE 1:** The automated item selection procedure (AISP) results based on various lower bounds.

Variable	Latent class		
Scaling criteria	0.4	0.5	0.6
B1, angry	1	1	1
B2, sad	2	4	3
B3, frustrated	1	1	3
B4, anxious	2	4	0
C1, neck pain	1	2	2
C2, headache	1	2	2
C3, sleepiness	1	0	0
C4, muscle stiffness	1	1	1
C5, increased heartbeat	1	3	0
C6, sweaty palm	1	0	0
C7, tired	1	0	0
C8, motion sickness	1	3	0
C9, difficulty in concentration	1	3	0
C10, back pain	1	2	0
C11, drawing sensation in body	1	1	1
C12, aggression	1	1	1
C13, stomach pain	1	3	4
C14, restlessness	0	3	4

**TABLE 2:** Statistics and scalability summary of scales with related items.

Latent	Variables	Statistics		Criteria	
		Mean	SD	*H*_*j*_	SE
1. Exhaustion	C3, sleepiness	2.05	1.14	0.42	0.50
	C6, sweaty palm	2.78	1.21	0.42	0.045
	C7, tired	1.88	0.94	0.43	0.047
*H* = 0.42, α = 0.63 λ_2_ = 0.63 *MS* = 0.65					
2. Angry	B1, angry	1.84	0.93	0.60	0.37
	B3, frustrated	1.87	0.99	0.51	0.047
	C4, muscle stiffness	2.24	1.10	0.64	0.034
	C11, drawing sensation in body	1.98	1.03	0.61	0.038
	C12, aggression	2.16	1.11	0.65	0.034
*H* = 0.60, α = 0.86 λ_2_ = 0.87 *MS* = 0.88					
3. Upper body pain	C1, neck pain	2.31	1.14	0.66	0.029
	C2, headache	2.38	1.14	0.60	0.040
	C10, back pain	2.25	1.13	0.60	0.041
*H* = 0.82, α = 0.81 λ_2_ = 0.81 *MS* = 0.82					
4. Physical tension	C5, increased heartbeat	2.91	1.14	0.56	0.034
	C8, motion sickness	3.22	1.16	0.56	0.033
	C9, difficulty in concentration	2.60	1.16	0.52	0.037
	C13, stomach pain	3.31	1.11	0.54	0.037
	C14, restlessness	2.97	1.17	0.55	0.036
*H* = 0.55, α = 0.84 λ_2_ = 0.83 *MS* = 0.84					
5. Bad feelings	B2, sad	2.5	1.19	0.57	0.046
	B4, anxious	1.99	1.02	0.57	0.046
*H* = 0.57, α = 0.68 λ_2_ = 0.68 *MS* = 0.70					

## Materials and Methods

The MSA is a non-parametric IRT that aimed at assessing unidimensional scales of dichotomous or polytomous items. The method section follows five subsections: scalability, the automated item selection procedure (AISP), local dependence, non-intersection, monotonicity, and invariant item ordering (IIO). This section will outline the mathematical formulation being used to estimate parameters’ estimates.

### Scalability

For all the incorporated methods, the scalability coefficients are used to describe evaluation of a variety of measurement properties including unidimensionality and local dependence (Meijer et al., 2014). Item-pair Scalability close to zero is an indication of much Guttman error while value of 1 indicates no Guttman error. The value could be written as


(1) $$H_{i\,j}=1-\frac{F_{i\,j}}{E_{i\,j}}$$

Where *F*_*ij*_ is the observed frequency of Guttman errors, while *E*_*ij*_is the expected frequency of Guttman errors. The interpretation of scalability scale coefficients varies as 0≤*H*≤1 could be as follows *H*≥0.5 corresponds with strong scale, 0.40≤*H* < 0.50 corresponds with medium scale; and 0.30≤*H* < 0.40 corresponds weak scale (Molenaar and Sijtsma, 2000).

In the MSA, items from the same Mokken scale should have an item scalability coefficient greater than 0.3 (Sijtsma and Molenaar, 2002), and *H*_*j*_ could be interpreted in a similar way as the discrimination parameter in IRT (Van Abswoude et al., 2004).

### Automated Item Selection Procedure

Selecting items from a larger set into clusters by measuring latent trait with enough discrimination power has been referred to the Automated Item Selection Procedure (AISP) (Sijtsma and Molenaar, 2002). The process could be seen as an alternative to factor analysis, employing H coefficient as its criterion for keeping or rejecting a set of items. Item quality has often been defined as the degree to which an item could precisely distinguish respondents, with low and high measurement values (Straat et al., 2013).

A set of items has been referred to a scale. The AISP select items clusters satisfying the Mokken’s scale definition (Mokken, 2011). The criteria are


(2) $$\rho_{i\,j}>0\,f\,o\,r\,a\,l\,l\,i\,t\,e\,m\,s\,p\,a\,i\,r\to c\,o\,v\,\left(X_{i},X_{j}\right)>0\to H_{i\,j}>0$$

(3) $$H_{i}\geq c>0\,f\,o\,r\,a\,l\,l\,i\,t\,e\,m\,i$$

From Equation 3, choosing a higher *c* is an indication that the AISP would choose items with higher power in terms of discrimination (Sijtsma and Molenaar, 2002). Also, higher *c* means a stronger scale. The method selects an item only based on correlation, and *H* coefficient, see Equations 2 and 3. The scalability coefficients *H*_*j*_play a role by separating items having low or high quality in relations to the test-score distribution. Scalability coefficients were used to assess items quality in a given set of items or AISP items (Sijtsma and Molenaar, 2002).

Based on the above discussion, scalability is summarized into three main equations: scalability coefficient *H*_*ij*_ for item pairs, item itself *H*_*j*_, and weighted average of J coefficients *H*. The item-pair scalability coefficient *H*_*ij*_ could be written as:


(4) $$H_{i\,j}=\frac{C\,o\,v\,\left(X_{j},X_{j}\right)}{c\,o\,v_{m\,a\,x}\,\left(X_{j},X_{j}\right)}$$

Where *cov*_*max*_(.) is maximum covariance which is employed by first sorting all covariates and then obtaining a pairwise variance (Van der Ark, 2007), and *Cov*(*X*_*j*_,*X*_*j*_) is the covariance between two items. The scalability coefficients have been used to assess item quality or a set of items in the AISP (Sijtsma and Molenaar, 2002). The above equation is equivalent to Equation 1.

Rest score of *R*_*(j)*_ is defined as *R*_*j*_ = *X*_+_−*X*_*j*_, and the item scalability coefficient *H*_*j*_ is written as:


(5) $$H_{j}=\frac{C\,o\,v\,\left(X_{j},R_{j}\right)}{C\,o\,v_{m\,a\,x}\,\left(X_{j},R_{j}\right)}$$

To achieve *R*_*j*_, all the diagonal of variance would be set as 0, so the sum of rows would not include that variable itself (*X*_+_−*X*_*j*_). And weighted average of the J coefficient as H, scalability coefficient, could be defined as (Mokken et al., 1986):


(6) $$H=\frac{\sum_{j=1}^{J}C\,o\,v\,\left(X_{J},R_{j}\right)}{\sum_{j=1}^{J}c\,o\,v_{m\,a\,x}\,\left(X_{j},R_{j}\right)}$$

It should be noted while *H*_*ij*_ define relationship between two items, *H*_*j*_ highlights an association of item j with its latent factor, while *H* express the accuracy.

The non-technical process could be summarized in few steps as highlighted by Mokken (2011). Start with pair of items i1 and j1, having the largest*H*_*ij*_. From the pool of k-2 items, j2 could be selected, which correlates positively with the previous two items, and its *H* fulfill the criteria of being greater than *c*, and maximize the *H* for the previously chosen items. In the next step, the same process would be implemented for j3 from the pool of k - 3. If items left to be selected, the AISP would try to find a scale to accommodate the remained items. The process would continue until none of the remaining items satisfy the criteria.

In MSA, items from the same Mokken scale should have an item scalability coefficient greater than *c* > 0.3 (Sijtsma and Molenaar, 2002), and *H*_*j*_could be interpreted in a similar way as the discrimination parameter in IRT (Van Abswoude et al., 2004). It should be noted that the criteria of *c* > 0.3 as a main criterion to check if a scale has a necessarily requirement to be considered in the analysis or not.

### Local Independence

Let j indexes item, and *X*_*j*_ be a polytomous item variable, and θ be the latent factor. Item scores are independent given θ if:


(7) $$P\left(X_{1}=x_{1},\ldots ,X_{J}=X_{j}|\theta \right)=\prod_{j=1}^{J}P\left(X_{J}=x_{j}|\theta \right)$$

In case of local dependence, the items have either positive local dependence (PLD), or negative local dependence (NLD).

After obtaining the results of the local dependence, the results would be used for removing those items having locally dependent item subset. Indices of *W*^(1)^,*W*^(2)^,and*W*^(3)^ are used to quantify the degree of dependence.

In summary, the method is based on the correlation across two items. The correlation is used for estimating W, while W is used for flagging functions. To use that correlation value for W, Fisher z-transformation would be employed to do hypothesis test about the value of the correlation coefficient between items X and Y, *r*_*ij*_, while transforming the sampling distribution to approximately normal. And that could be written as:


(8) $$\mu_{i\,j}=0.5\times \text{ln}\,\left(\frac{1+r_{i\,j}}{1-r_{i\,j}}\right)$$

The estimation of *W*^(1)^,*W*^(2)^,and *W*^(3)^ are very similar so here to conserve space we will only discuss *W*^(1)^.

*W*^(1)^ between two items of a and b, which could be written as:


(9) $$W_{a\,b}^{1}=\sum_{j\neq a,c}\sum_{x}P\left(Z<\frac{-\mu_{a\,j|c\,\left(x\right)}}{\sigma_{c\,\left(x\right)}}\right)$$

Where $Z<\frac{-\mu_{a\,j|c\,\left(x\right)}}{\sigma_{c\,\left(x\right)}}$ is cumulative density function (CDF), and $\sigma_{c\,\left(x\right)}=\frac{1}{\sqrt{N_{c}-3}}$ and *N*_*c*_ is number of observations.

Flagging function is based on W so values would be considered as an outlier when based on Tukey fence (Tukey, 1977), there would be *W* > *Q*_3_ + 3×(*Q*_3_)−*Q*_2_. It should be noted that the same flag function are used for all W. It is worth noting that the removal of any flagged item might impact the number of flags for other items (Straat et al., 2016). Thus, flagged items would be removed one by one until no flagged items remain. In summary, this method is based on transferred correlation across two items through Turkey method.

### Non-intersection

The process is employed after successful implementation of local independence. There are various methods for checking the non-intersecting assumption which follows (Van der Ark, 2007):


(10) $$\begin{array}{c} \mathrm{If}\mathrm{for}a\mathrm{fixed}\mathrm{value}\mathrm{of}\theta_{0}:P\left(X_{i}\geq x|\theta_{0}\right)\geq P\left(X_{j}\geq y|\theta_{0}\right),\\ \text{then}\\ \mathrm{For}\mathrm{all}\theta P\left(X_{i}\geq x|\theta \right)\geq P\left(X_{j}\geq y|\theta \right) \end{array}$$

And the above equation, could be written as a manifest variable of W.


(11) $$P\left(X_{i}\geq x|W=\omega \right)\geq P\left(X_{j}\geq y|W=\omega \right),forall\omega$$

Mokken shows that if Equation 10 holds, then we have (Mokken et al., 1986)


$$P\left(X_{i}\geq x,X_{k}\geq z\right)\geq P\left(X_{j}\geq x,X_{k}\geq z\right)$$

(12) f⁢o⁢r⁢z=1,…,m;i≠k;j≠k

$$P\left(X_{i}<x,X_{k}<z\right)\leq P\left(X_{j}<y,X_{k}<z\right)$$

(13) f⁢o⁢r⁢z=1,…,m,i≠k;j≠k

Where both parts in the left and right of the above equations are joint probabilities, which are saved in Jm × Jm matrices. The two matrices are *P*_+ +_ and *P*_−−_ being based on Equations 12 and 13, respectively. The two matrices are made in such a way that the comparison would be made based on the above equations: to make a comparison and to check the above inequality, the differences between values are estimated.

In summary, the results are mainly based on created two matrices. The violations are identified based on a comparison against the minimum acceptable values of the differences, or how much the two sides of the inequalities in the above equation are varied.

Another important aspect is the process of conversion of the scores into steps. The main objective of those steps is to order the items based on the levels of popularities, or repetition of various categories. The process is checked against the minimum criteria values, and if it is passed the minimum values then it is flagged as violations. It should be noted that the included results are based on method of pmatrix (Molenaar and Sijtsma, 2000).

### Monotonicity

Monotonicity is similar to invariant item ordering (IIO) in terms of the use of grouping. The significant difference is that IIO was dealing with two items at a time while monotonicity deals with an individual item. In other words, for each item, instead of working on the item’s category it would loop over the groups: using the rest score for creating groups.

In summary, [*X*_*j*_ = 1|*R* = *r*−1]≤*P*[*X*_*j*_ = 1|*R* = 1], we have


(14) $$\left\{ \begin{array}{ccc} P\left(X_{j}=1|R=r-1\right)= & & \\ \int P\left[X_{j}=1|R=r-1,\theta \right)dF\left(\theta |R=r-1\right) & & \\ \leq \int P\left(X_{j}=1|R=r,\theta \right)dF\left(\theta |R=r-1\right) & & \\ \leq \int P\left(X_{j}=1|R=r,\theta \right)dF\left(\theta |R=r\right) & & \\ \leq \int P\left(X_{j}=1|R=r\right) & & \end{array}\right.$$

So, whether there is a violation or not is checked from the above equations where the integral includes the sum of process to estimate the probability of X being greater than values based on groups number (e.g., *X*≥1) and dividing the cumulative sum by frequency of the total number in each group. The check, then, would be achieved by testing the violation against the minimum violation limit like IIO.

### Invariant Item Ordering

It should be noted that the comparison will be based on one pair of variables at a time for each scale. The minimum value for checking the violation was set as m × 0.06, where m is number of categories minus value of 1 (Molenaar and Sijtsma, 2000). Due to a pairwise comparison, two varied values of i and j are created, e.g., 1 and 2. The method is based on the manifest invariant item ordering (MIIO) so the process would be explained for this method.

Matrix of R will be created. That is the sum of scale of all considered items except for the first column. Two items would be picked up. Adjustment for group would be made until the group size exceeds a minimum value (Molenaar and Sijtsma, 2000). Now results would be given based on numbers of assigned groups. A comparison would be made not based on the items’ categories but the membership. For instance, the same item might have different response probabilities (Sijtsma and van der Ark, 2017), and those variations should be taken into consideration. Thus, groups would be used in the MSA analysis allowing for those scale properties in various groups to be evaluated.

The individuals were ordered by groups as the ordering of various items, based on group mean scores, does not indicate that the ordering holds at individual level (Ligtvoet et al., 2010). Thus, when items have IIO at the group levels, then their structures could be assumed to be valid at the individual’s levels. The model is implemented in R using Mokken package (Van der Ark, 2007).

## Results

The analysis of the instrument would be presented in the order of: 1. AISP, 2. scalability (along with reliability measures), 3. Local dependence 4. Non-intersection, 5. Monotonicity, and 6. IIO. Although most of past study did not consider the non-intersection as a criterion to investigate the suitability of an instrument, that method also was employed in this study. Each section will follow interpretation of results and proposal of some modification. At the end, based on all the observations, the conclusion will be made.

### Automated Item Selection Procedure

The AISP was conducted before any measure to highlight the scales with related items. The objective of the AISP is highlighted as a selection of many sufficiently discriminating items as possible in each class (Sijtsma and Molenaar, 2002). First, we used automated item selection procedure, and then based on the next subsection, or scalability, whether or not all the items have the psychometric quality needed for selection of the finals scale was determined.

Various lower bound, c, were considered and the items within each scale were compared to come up with better scales, in terms of interpretability and reliability. However, values less than 0.4 results in identification of only a single scale, and c value of 0.4, resulted in mostly a single latent and few latent with only few items see Table 1.

As can be seen from Table 1, apart from the lack of fit, which the next section will discuss, the first latent classes based on lower bound of 0.4 include only mainly a single class, while the lower bound of 0.6 resulted in classes with two latent classes with very low numbers of scales with only 2 items. The further discussion of this section would be presented along with the next subsection.

### Scalability

The first considered step after the AISP is to make sure weak items would not slip into the scale, while other methods in next steps would be employed for further diagnosis. Items making a scale with *H* < 0.3 would be considered unscalable (Sijtsma and Molenaar, 2002). Also, non-negative correlation is expected between a variable and an item (Strout, 1990). The process is measured for each scale separately after conducting AISP. The scalability of item pairs, Scalability of the item, and Scalability of the entire scale were estimated and considered as *H*_*ij*_, *H*_*i*_, and H, respectively.

To make Table 2 simple, the item scalability coefficient *H*_*j*_, along with scale scalability coefficient of H were only included. Recall, values of H vary from 0 to 1, where *H*≥0.50 is a strong scale, and H between 0.4 and 0.5 is a medium scale. As discussed, and based on Table 1, considering *c* = 0.4 resulted in many *H*_*j*_ being less than 0.4, highlighting a low correlation between an item and *R*_*(j)*_. This highlight that the item is unlikely to belong to the assigned scale. Very low values also were found for *H*_*ij*_ highlighting low correlation across the items within a same scale. A similar issue, with less severity was observed for *c* > 0.6.

Based on the obtained results of *c* = 0.5, the results show that the scales coefficients H_*j*_ vary from 0.64 for the second scale to 0.42 for the first scale, and no concern could be observed for the scales, see Table 2. The lower part of Table 2 presents the Guttman’s lambda-2 (*λ*_*2*_), Molenaar-Sijtsma (MS) method, Cronbach’s alpha (α), and total of H. It should be highlighted that the traditional method of Cronbach’s alpha highlights no issue with our instrument.

As can be seen, all the latent classes provide an acceptable range with the minimum of H related to the first latent class (0.42). However, as all latent classes are within fair or good range: all of them would continue to be evaluated for the next stage. The results of the means of the items, show that, with the exception of a few items for the 4th class (motion sickness and stomach pain), the respondent mostly agree with the experienced feelings.

The means of the items illustrate the degree of feelings experienced by commuters while facing delay. As can be seen from the means, while the commuters are mostly in agreement with experiencing anger and frustration while facing delay, the highest disagreement was observed for motion sickness. Descriptive statistics of parameters in terms of mean and standard deviation (SD), and the scalability coefficient (*H*_*j*_) and related standard errors (SE) are presented in Table 2.

### Local Independence

Local independence measures whether individual item scores are independent given θ: respondents being higher on θare expected to obtain higher item scores (Sijtsma and van der Ark, 2017). Thus, items measuring the same θare expected to correlate positively, while respondents vary in regard to θ. However, by selecting a subgroup of respondents having same θ value, the relationship or correlation across the items vanishes (Sijtsma and van der Ark, 2017). W’s would be used as indices to flag locally dependent item pairs.

For this section, due to the limitation of computation of a covariance for number of items, being less than 4 within a scale, the process was employed only on two scales with more than 4 items in each scale. For the second scale (angry), a large value of W1 for C11 (draw sensation), and C12 (aggression) and also between B1 (angry) and B3 (frustrated) highlight a high positive correlation across the items, flagging those items as being locally dependent. That might be an indication of highlighting some of the items which might need to be revised.

After few trial and error runs, removal of B1 resulted in no violation of local dependence assumption. It should be noted that while removal of B3 was also acceptable, removal of C11, and especially C12 did not resolve the problem, still resulting in unacceptable scale.

### Non-intersection

**FIGURE 2:**
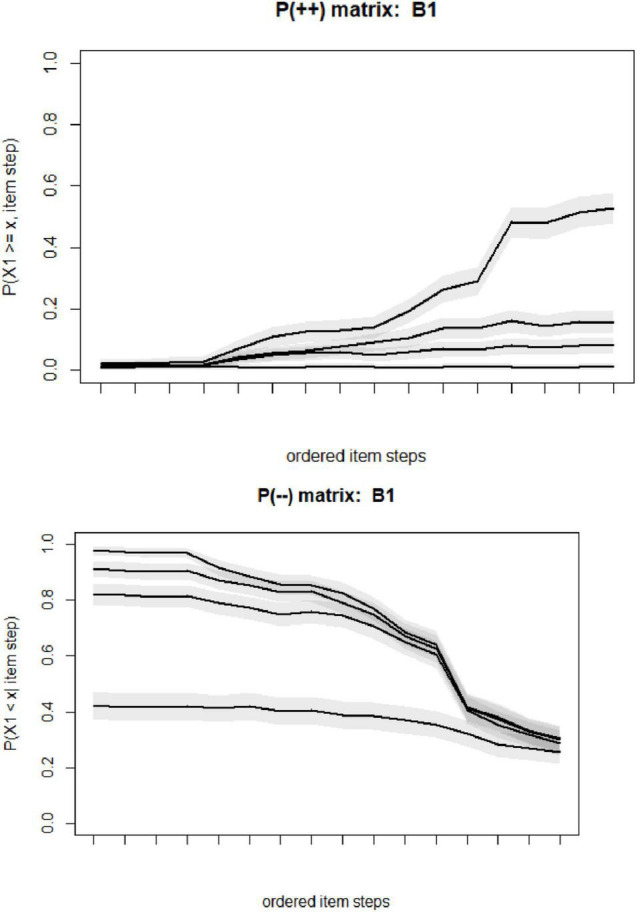
Results of non-intersecting diagnostic for item B1.

Non-intersecting highlight whether the item step response function intersect or not. The output highlight that all items have some violations greater than 0.03. However, based on the figures and violation values, it is observed that the violations were not extreme, being within the range of 0.04. The plots of P++ and P−− were presented for B1, as an example, in Figure 2.

The computed values of P(++), and the P(−−) matrices are each (Jm × Jm) or 16 × 16, and the Figure 2 is depicted for each P(++), and P(++). Few observations should be noted from Figure 2. The extreme left on the horizontal axis is the least popular item step of X3 = 4, while the extreme on the right is related to X2 = 1.

There are a total of 20 steps [(5-1) × 5]. The steps are highlighted as ordered *I*_1_,…,*I*_20_.Here for instance, the first figure is related to X1 (B1), the top figure is related to *P*(*X*_1_≥1,*I*_1_),…,*P*(*X*_1_≥1,*I*_20_), and the lowest line is *P*(*X*_1_≥4,*I*_1_),…,*P*(*X*_1_≥4,*I*_20_). Again, although this diagnosis measure has not been highlighted as a required step, this diagnostic was provided to present a further insight.

It should be noted that Item response function (ISRFs) was used for rating scale categories. For k categories we have k-1 ISRF’s (steps) reflecting the response probability for a given category or higher across latent variable. Non-intersecting ISRF’s highlight that the conditional probability for the category k rating or higher for item i has similar relative ordering across all latent variables’ values.

### Monotonicity

**FIGURE 3:**
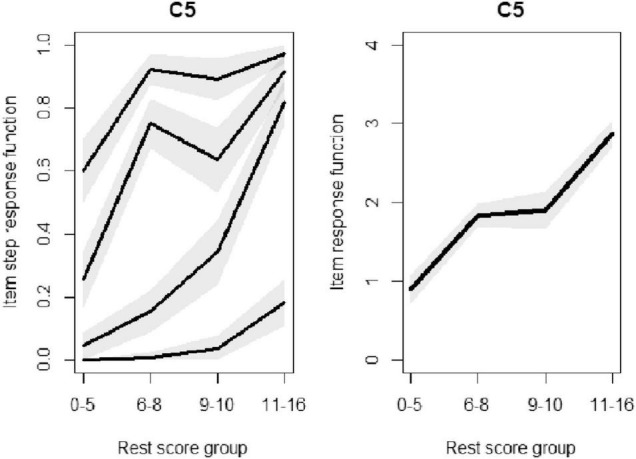
Violation of monotonicity evaluation for C5, as an example.

There are four groups along with four probabilities for each item. So, based on Equation 14, there would be plotted conditions on R, rest score group, and for m-1 categories. As a result, we expect to have 5-1 plots for each measurement, see panel on the left side of Figure 3. To ensure the monotonicity, it is expected that the probability will increase by an increase in the number of groups. If the assumption does not hold true, there is a violation of the monotonicity.

The monotonicity was employed on all scales. Regarding the first scale of exhaustion, C3 (sleepiness) was found to be in violation of monotonicity. For scale number 2, angry, no violation of monotonicity could be observed. Also, for the 3rd scale, upper body pain, no concern could be observed. For the 4th scale, C5 (increased heartbeat) is in violation of monotonicity. After removing C5, a new violation was observed for C14 instead. However, removing C13, instead of C5, resulted in no violations of manifest monotonicity, or having violation greater than minimum threshold value. No violation could be observed also for the last scale with two items: it could be concluded that the relative ordering of the respondents in answering the multiple choices is consistent.

For instance, for the forth scale, physical tension, for item C5 monotonicity issue was observed, for P(X = 1) and P(X = 2), which could be seen from Figure 3. Figure 3, on the left, highlight two violations, decrease for *P*(*X*_*C*5_≥1|*R*_−*C*5_ϵ{6,7,8} and P(*X*_*C*5_≥2|*R*_−5_ϵ{6,7,8}. In other words, and based on the results, while P(X = 1) increases from the first group to the second group, it decrease from the second group to the third group.

### Invariant Item Ordering

It has been argued that IRFs for real data often do not exhibit IIO but many intersections might occur because item ordering might vary greatly across various θ (Sijtsma and van der Ark, 2017). The idea for the IIO is based on items that do not show intersecting IRFs have an invariant item ordering (Sijtsma and Hemker, 1998).

There are three groups on the horizontal axis, rest score group size, while the vertical line is the expected values, item response function of a pair of items. It should be noted that the number of rest score group is dependent on numbers of observations. No violation could be observed for the first scale.

**FIGURE 4:**
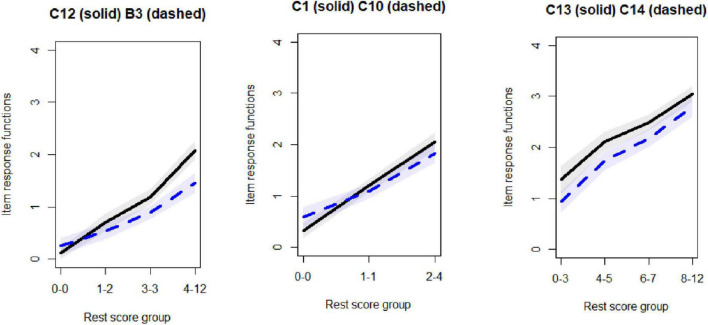
Invariant item ordering evaluations of few items, violations of invariant item ordering (IIO) for the two figures in the left, while no violation for the utmost in the right.

While no violation of the IIO was observed for items in the first scale, violation could be observed for B3 and C12 for the second scale, see Figure 4, the left panel. However, after removal of B3 no longer any violation could be observed. Moving to the third scale, violation was observed across C1 and C10, and also across C1 and C2. However, for this scale due to having a low number of items, no item removal could be conducted for addressing the IIO.

Regarding the fourth scale, no violation could be observed across any pairwise comparison of the items. The last scale has only two items so no comparison could be made. From the furthest plot in the right side of Figure 4, it can be observed that no violation of IIO could be observed as the IRFs have similar relative ordering across the range.

For several reasons only evaluation was considered for scale with number of items greater than 3. First, it is not applicable to choose IIO for scale with less than three items. Monotonicity was concerning for few of those low number scale, being an indication that low number scales are not well-suited scales. In addition, local dependence is challenging for scale less than three as there is the minimum required sample size of the conditioning variable to compute a covariance. In addition, there is a lack of interpretability for scale with very low numbers of items. So, we would consider only two scales with enough numbers of items.

## Discussion

The quantitative assessment of various psychological impact of public transport could be evaluated by implementing instruments, which measure individual’s experiences of those negative impacts. This study evaluated the construct validity, and related reliability of two subscale, psychological and physical, related to stress measurement.

In this research, we provided the MSA method, with less restrictive assumption compared with the IRT, for evaluation of the instrument. Specifically, the focus was on key criteria of the MSA including monotonicity, scalability, and invariant ordering. In addition, some key measurements such as local dependency, and non-intersection were considered. The AISP was employed first based on various threshold values of c to determine which scale is better to be considered. The c level of 0.3–0.4 resulted in many items falling below *H*_*i*_ coefficients of 0.3. On the other hand, c value greater than 0.6 resulted in an identification of many scales, where many scales only incorporate 1 or 2 items. Based on interpretability, a comparison was made across *c* = 0.5 and *c* = 0.6, resulting in a better interpretability for *c* = 0.5. The automated scaling divides the instrument into 6 subscales, while some scales incorporate only 2–3 items. In terms of scalability all scales had the minimum requirements needed for the MSA. Although concerns were raised regarding the local dependence criteria, removal of the item of B3 resulted in satisfactory results.

On the other hand, Monotonicity highlights a concern regarding the 4th latent, by highlighting violations across items C13 and C5. However, removal of C13 resulted in a satisfactory result. Regarding the IIO assumption, again, removal of B3 resulted in a satisfactory result for this assumption. Also, for low item category, the items were found to violate the necessary assumptions, or the tests were unable to be employed due to low number of items.

The last step is to modify the items experiencing violations of scalability. The sensitivity of scalability to the problems of instruments have been discussed in the literature (Wind, 2021). As the revision could not be readministered due to changes in circumstances, the items could be excluded from the analysis. Those items include C13 (stomach pain) and C5 (increased heartbeat).

In summary, although the focus of this study was on two scales with enough number of items, greater than 3, the other small scales were found to be in violations of the assumptions or incapability of the model to be employed due to their shortcomings of number of items. Again, as revision and conduction of the instrument is not practical, those items might be reconsidered in the future study as a single scale and the suitability of that scale should be reevaluated. That is based on past literature suggesting the consideration of rejected items as a new candidate scale, and checking for construct validity to make sure they fulfill the MSA criteria (Stochl et al., 2012).

One limitation of this study is that we did not investigate the causes of problems with those items including stomach pain and increased heart rate. Future studies should take advantage of additional follow-up and more investigation through interviews with commuters to understand the implications of those shortcomings. This could help the researchers to better connect the lack of strength for few items and real implication of those findings.

However, modification of the items showed that by removing stomach pain and increased heart rate our instrument is suitable based on the MSA criteria. More studies are needed to consider the use of non-parametric process of Mokken scaling for evaluating the quality of the instruments, especially on transportation problems.

## Conclusion

Compared with the parametric IRT, the MSA uses the non-parametric IRT to employ a less restrictive assumptions regarding the data, and it investigates the population of interest in more depth (Junker and Sijtsma, 2001). In addition to three main assumptions of the MSA, including unidimensionality, monotonicity, and local dependence, measurements such as scalability was considered to provide a deeper perspective regarding the performance of the model. The results highlighted concerns for two variables in terms of scalability. Recommendations were proposed to address those concerns.

In light of the findings of this study, our instrument could be implemented in other studies by applying modifications on some items by rewording or providing more clarification. Also, adjustments could be made by changing the response choices. Special attention should be given in future studies for scales with low number of items.

## Data Availability Statement

The original contributions presented in the study are included in the article/supplementary material, further inquiries can be directed to the corresponding author.

## Author Contributions

MR, CV, KC, and FF: methodology and writing. All authors contributed to the article and approved the submitted version.

## Conflict of Interest

The authors declare that the research was conducted in the absence of any commercial or financial relationships that could be construed as a potential conflict of interest.

## Publisher’s Note

All claims expressed in this article are solely those of the authors and do not necessarily represent those of their affiliated organizations, or those of the publisher, the editors and the reviewers. Any product that may be evaluated in this article, or claim that may be made by its manufacturer, is not guaranteed or endorsed by the publisher.
